# Supplementary material for: Health professionals’ readiness to implement electronic medical recording system and associated factors in public general hospitals of Sidama region, Ethiopia
Source: PLoS One. 2022 Oct 18;17(10):e0276371. doi: 10.1371/journal.pone.0276371 (PMC9578591; doi:10.1371/journal.pone.0276371)
Supplement: Attachment — Submitted filename: Response to Reviewers.docx [file pone.0276371.s002.docx]

We would like to take a moment to thank the Editor and the peer reviewers for the constructive evaluation of our paper. We have corrected the manuscript as per the comments provided and we hope the manuscript meets the requirement for publication.

Sincerely,

Kibruyisfaw Weldeab Abore

**Response to Academic editor**

1. Please ensure that your manuscript meets PLOS ONE's style requirements, including those for file naming.

We have edited authors contributorship as per the journal requirement. We have also edited the figure files using PACE and revised the file names to suit the journal.

2. We note that the grant information you provided in the ‘Funding Information’ and ‘Financial Disclosure’ sections do not match. When you resubmit, please ensure that you provide the correct grant numbers for the awards you received for your study in the ‘Funding Information’ section.

We have amended the information on both sections

3. Upon re-submitting your revised manuscript, please upload your study’s minimal underlying data set as either Supporting Information files or to a stable, public repository and include the relevant URLs, DOIs, or accession numbers within your revised cover letter.

Thank you for the comments. We have availed the minimal data set as a supporting information.

**Reviewer 1**

**Abstract:** The result doesn’t reflect anything from qualitative section. No need to mention the name of Software used in data analysis

We amended abstract section to include results from the qualitative analysis and we have removed the name of the software used in data analysis from the abstract.

**Introduction:** Kindly reflect how EMR system could contribute to better data management and what kind of health data are mostly aimed to handle by EMR system in Ethiopia

We have amended the manuscript to explain the kind of health data intended to be handled by EMR and the role of EMR for better data management.

**Methods:** Provide justification why p value less than 0.25 was considered as candidate for multivariate Logistic regression to determine predictors of readiness. On what basis were the professionals who scored ≥ 50% on literacy questions classified as computer literate? Mention the process of qualitative data analysis. Focus on preconceptions and meta-positions for qualitative data analysis process

Although different scholars use p-value < 0.2 for including predictors in to the multivariable logistic regression based on Hosmer and lemshow applied logistic regression recommendation, we used p-value< 0.25 to include as many predictor variables as possible in the multivariable model so as to ensure adjustment of confounders. Regarding computer literacy, we classified those participants with the average computer literacy score or above as literate. The questions were composed of questions which assessed basic computer skills of individuals. Pertaining to the process of qualitative analysis we have included details in the method and material section to meet reviewers comment.

**Results and discussions:** Although the result fairly complies with the quantitative analysis, it seriously lacks the qualitative findings. There are only two sentences from the participants which do not show the strength of the qualitative data analysis. What theme was developed from the thematic analysis? The discussion should be more detailed and should focus on validity and reliability for quantitative findings and transferability for qualitative findings

We have added a subsection labeled perceived barriers for readiness to implement EMR to address themes and subthemes that emerged during thematic analysis.

**Recommendations and conclusions:** could be separate sections.

We have amended this section in to two separate sections.

**Reviewer 2.**

**Abstract:**

1. There are a few grammatical and punctuation errors in the manuscript. This should be addressed in the revised version.

Thank you for the comments. We have amended the manuscript based on the comments.

2. “Electronic medical recording system is one of the information technologies that have a proven benefit to improve the quality of health service.” It will be “has a proven benefit”.

Thank you for the comment. We have addressed the comment

3.“A hospital based cross-sectional study design”; remove the term “hospital based”, as in the subsequent texts, it is mentioned that the data was collected from the hospital.

Thank you for the comment. We have addressed the comment

**Introduction:**
4.“Globally, less than half of the world countries has adopted a national EMR system according to a 2016 WHO report, although there have been improvements over the past decade [1, 8]. The report had also shown disparities in the adoption of the system among countries. A 2013 report indicated that more than 90% of primary care physicians in New Zealand, Netherlands, United Kingdom, Australia, Sweden, and Norway used EMR system [9].” These are old statistics.Please report the updated statistics.

Thank you for the comments. We have amended the section to include more updated information
5.MOH: use the full form of its first appearance.

Thank you for the comment. We have addressed the comment

6.“There are few published studies done in Ethiopia to assess health professional readiness and there was no study conducted in the region to best of the investigators knowledge.” Use citations.
Thank you for the comment. We have addressed the comment in the introduction section.

7.Mention the objective of the study at the end of the introduction.

Thank you for the comment. We have addressed the comment at the end of the introduction section.

**Methods and materials:**
8. “Based on up to date information there are four general hospitals in the region namely Adare, Yirgalem, Bona, and Leku which are found 275 km, 325km, 392km, and 306 km away from the capital Addis Ababa respectively.” Use citations.

Thank you for the comment.
9. The authors mentioned G.C at the end of the date. What does it mean?

Thank you for the comment. It means Gregorian calendar while in Ethiopia we use Ethiopian calendar ( E.C). we have removed all references of G.C from the manuscript.
10. What are the types of health professionals included in the study? It should be mentioned in the study population section.

Thank you for the comment. All health professionals working in the selected health facilities were considered the study population. Meanwhile, the details of the professional category were addressed in the sociodemographic part of the result.

11. “12 key informants” from how many hospitals? From any single or from all hospitals?

A total of 12 key informants were selected from all hospitals. We have amended the manuscript to address the concern.
12. In the logistic regression, it will multivariable analysis. Not multivariate analysis, as there is dichotomous dependent variable.

Thank you for the valuable comment. We have addressed the comment at the method and material section and result section.

13. Operational definitions should be placed before data processing and analysis section.

Thank you for the comment. We have placed operational definitions before data processing and analysis section.

**Results**
14. “Of the respondents 192 (70.1%) have a first degree while 21(7.7%) health professionals had a second degree and above.” What are first degree and second degree? Not clear. Explain in the methods section or in the result interpretation.

Thank you for the comment. We have addressed the issue in the result section. We have changed it to bachelor degree and Masters degree and above.
15. In table 1, what is HIT profession?

It means Health information technology professionals. We have amended it in the result section
16. In table 1, What does mean by degree and second degree professions?

Thank you for the comment. We have addressed the issue in the result section. We have changed it to bachelor degree and Masters degree and above.
17. “among the study participants, only 31 (11.3%) have previous training while only 63 (23%) had previous experience.” Write in the past tense.

Thank you for the comment. We have addressed the issue in the result section.
18. In table 2, the variables ‘previous training’ and ‘previous experience’ are not clear. What training and what experience?

Thank you for the comment. We have addressed the issue in the result section to previous EMR experience and previous EMR training.
19. In table 3, p value placement of ‘duration of employment’ is not correct. The authors put value only for unadjusted analysis. Why not for adjusted analysis? Put p value for both the analyses, or indicate it using asterisk.

Thank you for the comment. We have addressed the issue in the result section and we have indicated significant p-values using asterisk.

20. It is not clear how the authors adjusted the readiness of EMR use in the regression model. It should be adjusted by including some important demographic variables also, i.e. age, sex, educational status, profession etc.

Thank you for the comment. Although in the result section we have only displayed those with significant p-value on adjusted analysis, we did the multivariable logistic regression including variables from sociodemographic characteristics and organization and technical factors to adjust for confounders. If needed we can provide the detailed crude analysis and adjusted analysis results.

**References**
21. For most of the citations, there is no doi or url in the references.

Thank you for the comments. We have updated the references to include doi and url
22. There is no data as supplementary file.

Thank you for the comments. We have included minimal data as a supplementary file
