# Supplementary material for: Health professionals’ readiness to implement electronic medical recording system and associated factors in public general hospitals of Sidama region, Ethiopia
Source: PLoS One. 2022 Oct 18;17(10):e0276371. doi: 10.1371/journal.pone.0276371 (PMC9578591; doi:10.1371/journal.pone.0276371)
Supplement: Attachment — Submitted filename: response to reviewers.docx [file pone.0276371.s003.docx]

We would like to take a moment to thank the Editor and the peer reviewers for the constructive evaluation of our paper. We have corrected the manuscript as per the comments provided and we hope the manuscript meets the requirement for publication.

Sincerely,

Kibruyisfaw Weldeab Abore

**Response to academic editor**

Please review your reference list to ensure that it is complete and correct. If you have cited papers that have been retracted, please include the rationale for doing so in the manuscript text, or remove these references and replace them with relevant current references. Any changes to the reference list should be mentioned in the rebuttal letter that accompanies your revised manuscript. If you need to cite a retracted article, indicate the article’s retracted status in the References list and also include a citation and full reference for the retraction notice

Dear editor,

Thank you for taking the time to review our manuscript and for the constructive comment. After reviewing the reference list, although we have the booklet at hand, we noticed that one of the references booklets from ministry of health Ethiopia had no database to which readers could access the document. Therefore, we have removed this reference and updated it with a roadmap booklet that can be accessed from ministry of health E-library.

**Reviewer 1:** All the comments have been addressed. Therefore, I suggest to accept the manuscript. Furthermore, the underlying dataset could be checked, and probably a single excel sheet would be more aesthetic for the interested readers/researchers. If possible, provide the coded transcripts as the minimum underlying dataset for the qualitative part. The statistical issues could be re-checked by the expert before publication.

I would like to extend my sincere gratitude on behalf of all authors for the constructive review we received and for the support towards the publication.

**Reviewer 2:** I would like to make a suggestion to the authors that in Table 3, they should include a footnote stating for which variables they accounted for adjusting their analysis.

I would like to extend my sincere gratitude on behalf of all authors for the constructive review we received and for the support towards the publication. We have amended the manuscript to include a foot note on Table 3 for the variables accounted for in the multivariable logistic regression.
